# Lynch Syndrome: An Update of Underlying Molecular Mechanisms, Phenotypes and Methods to Classify Variants of Uncertain Significance

**DOI:** 10.3390/biomedicines14061312

**Published:** 2026-06-09

**Authors:** Pedro Rodrigues, Paulo Matos, João Gonçalves, Peter Jordan

**Affiliations:** 1 Department of Human Genetics, National Institute of Health Dr. Ricardo Jorge, 1649-016 Lisbon, Portugal; paulo.matos@insa.min-saude.pt (P.M.); joao.goncalves@insa.min-saude.pt (J.G.); 2 Comprehensive Health Research Centre (CHRC), NOVA Medical School, Universidade NOVA de Lisboa, 1169-056 Lisbon, Portugal; 3 BioISI—Instituto de Biossistemas e Ciências Integrativas, Faculdade de Ciências, Universidade de Lisboa, 1749-016 Lisbon, Portugal

**Keywords:** Lynch syndrome, mismatch repair, variant of uncertain significance, hereditary cancer, functional assays

## Abstract

In 2022, colorectal cancer (CRC) was the third most common type of cancer worldwide and the second most common in Europe. CRC ranked as the second leading cause of cancer-related deaths both worldwide and in Europe, with 904,019 and 247,966 deaths, respectively. The majority of CRC cases are sporadic (60–75%); however, 10–35% of CRC are estimated to result from the interaction of heritable and environmental factors. Among these, 5–6% are caused by inherited variants in genes that predispose to the development of CRC. Among the known inherited causes, Lynch Syndrome (LS), formerly known as Hereditary Nonpolyposis Colorectal Cancer (HNPCC), is the most frequent and accounts for approximately 3% of all CRC. Here we review and update on multiple aspects of LS in the context of CRC, including its genetic and molecular basis, current guidelines for molecular screening and variant classification. Furthermore, we review functional assays that have been used to determine the biological impact of genetic variants of uncertain significance (VUS) and discuss future perspectives in the field.

## 1. Introduction

In 2022, colorectal cancer (CRC) was the third most common type of cancer worldwide, accounting for 1,926,425 cases (9.6%), and the second most common in Europe, with 538,584 cases (12.0%). Regarding mortality, CRC ranked as the second leading cause of cancer-related deaths both worldwide and in Europe, with 904,019 (9.3%) and 247,966 (12.5%) deaths, respectively [1,2].

The majority of CRC cases (60–75%) are sporadic [3,4] and among them, approximately 70% develop from adenomatous polyps, 25–30% arise from sessile serrated lesions (SSLs) through the serrated pathway, and a small proportion (1–2%) develop from inflammatory bowel diseases [5,6,7].

**Figure 1:**
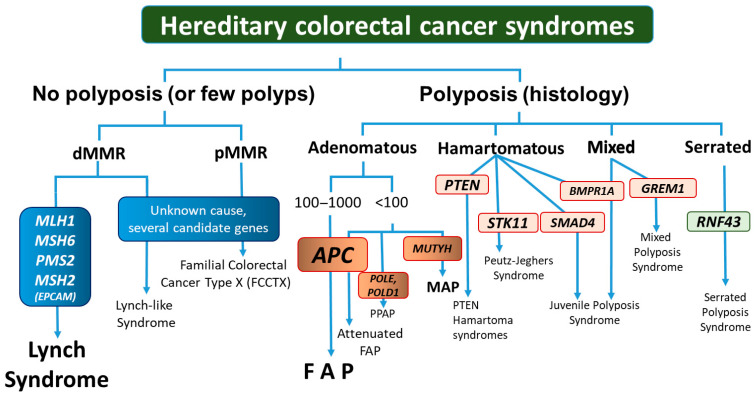
Overview of the most frequent hereditary colorectal cancer syndromes and affected genes. Syndromes presenting polyposis are subdivided according to the indicated polyp histology, while syndromes without or few polyps are differentiated according to their mismatch repair (MMR) status. Coloured ellipsoids indicate the affected genes and arrows the syndrome name. Indicated are the 3 most frequent syndromes in bold, and the number of polyps usually detected in adenomatous polyposis patients; d = deficient, p = proficient, FAP = familial adenomatous polyposis, PPAP = Polymerase Proofreading-Associated Polyposis, MAP = MUTYH-associated polyposis.

The remaining CRC cases are associated with familial factors (10–35%), both environmental and genetic, including several hereditary CRC syndromes (HCRCS) that account for 5–6% of all CRC cases and are caused by inherited variants in well-characterized tumour suppressor genes [8,9,10,11,12]. HCRCS can present with or without polyposis (or only few polyps). Lynch Syndrome (LS) is the most common cause of HCRCS, accounting for at least 3% of all CRC cases [13,14], followed by familial adenomatous polyposis (FAP) and *MUTYH*-associated polyposis (MAP) (see Figure 1).

LS presents without polyposis (or less than 15 polyps) [14,15]. LS-associated tumours are frequently mucinous adenocarcinoma, followed by other adenocarcinoma types, including medullary or rarely signet-ring cell adenocarcinoma [16,17,18]. The mucinous subtype is characterized by extracellular mucus comprising more than 50% of the tumour volume [19].

### 1.1. Early-Onset Colorectal Cancer

For the identification of LS patients, age of onset and family history are important criteria. However, a recent rise in CRC incidence among younger adults, also referred to as early-onset colorectal cancer (EOCRC), under 50 years of age, has underlined the need to distinguish early-onset sporadic disease clearly from hereditary predisposition.

A recent study, based on data from 50 countries available in the IARC database, concluded that the incidence of EOCRC is rising in 27 of the 50 countries analyzed [20], with some countries having doubled the incidence over the past two decades [21,22,23,24,25]. The rising incidence of EOCRC, is not exclusive to high-income Western countries but also occurs in Asia, Latin America, and the Caribbean [25]. A globalized food supply with increased consumption of ultra-processed red meat resulting in exposure to carcinogenic N-nitroso compounds, associated with a rise in obesity rates as a recognized risk factor for CRC, may both contribute to the rising incidence of EOCRC [20,26,27].

A recent genome-wide association study (GWAS) involving 6176 patients and 65,829 controls, identified two novel CRC risk loci (rs186107317 at 1p34.1 and rs9991540 at 4p15.33) associated with higher body mass index levels and metabolic alterations [28]. Additionally, Diaz-Gay et al. conducted a study involving 981 CRC cases, from more than 11 countries, and found that EOCRC patients revealed 3.3 times more common variations in the single-base substitution SBS88 and ID18. These are typically caused by the bacterial toxin and mutagen colibactin [25]. The life-style-dependent exposure to colibactin-producing bacteria from childhood onwards might be one of the factors that contribute to the increasing incidence of EOCRC. Therefore, despite the younger age of onset, it appears that most cases are sporadic and not associated with HCRCS [20].

### 1.2. Scope and Objectives of This Review

In this review, we present an update on the current knowledge of the molecular genetics and biology of LS, and novel developments concerning guidelines for the identification, interpretation and classification of germline variants. We highlight how evolving molecular tools and functional assays enforce the interpretation of mismatch repair variants of uncertain significance and improve clinical decision-making.

## 2. Materials and Methods

In order to provide an updated overview of LS, a literature search was performed on 25 November 2024 and updated again on 1 October 2025. NCCN guidelines for CRC screening were revisited on 2 March 2026. The public databases PubMed, ChatGPT (GPT-4o, OpenAI, San Francisco, CA, USA) and Google were used as search tools to identify relevant scientific papers, guidelines from professional societies and organizations, namely ClinGen, NCCN and CanVIG UK (see reference list for access information), and data from the clinical databases InSiGHT and ClinVar. The keywords used to collect information were Lynch Syndrome, Hereditary Nonpolyposis Colorectal Cancer, HNPCC, Colorectal Cancer, Microsatellite instability, Mismatch repair, MMR, MMR functional assays, MLH1, MSH2, MSH6, PMS2 and EPCAM. From this primary search result, relevant publications were selected by screening titles and abstracts, taking into account the quality, methodology, and relevance of the articles. Search results on functional assays were grouped together by type of assay, and then by organism or cell type employed.

## 3. Lynch Syndrome

### 3.1. A Few Historical Milestones

The history of the syndrome dates back to 1895 when Aldred Warthin began collecting information on the first LS family, later designated as Family G, which was affected by multiple cancers, with the colorectum, stomach, and uterus being the most prominent. Along with data from three other families (F, P and S), Warthin published his findings in 1913 [29]. He observed that the disease segregation of multiple cancers followed an autosomal dominant inheritance pattern. Later on, Hauser and Weller also contributed to Warthin’s work on LS [30].

In 1962, Henry Lynch began studying a family from Nebraska, which he referred to as Family N, and identified similarities with Family G. A few years later, in 1966, along with findings from another family in Michigan (Family M), Lynch and his colleagues published the pedigrees of both families, demonstrating an autosomal dominant inheritance pattern, as previously described by Warthin [31]. The first designation of LS was proposed in 1971 under the name Cancer Family Syndrome (CFS) [30].

LS was recognized as a hereditary cancer syndrome in 1993 due to the work of Peltomäki et al., who mapped the first genetic locus related to LS [32], along with Fishel et al. and Leach et al. who identified that pathogenic variants in *MSH2* segregated with affected members of LS families [33,34]. In the same year, microsatellite instability (MSI; described in more detail below) was also linked to LS [35]. The remaining LS loci, *MLH1* and *PMS2*, were identified in 1994 [36,37,38], while *MSH6* was identified in 1997 [39].

These major characteristics of LS still hold today: MSI due to loss of expression or function of one of four MMR proteins (MLH1, MSH2, MSH6, or PMS2). Besides pathogenic variants in these 4 MMR genes, LS can be caused by large deletions comprising the 3′ end of *EPCAM* [40], which is located less than 15.5 Kb upstream of *MSH2*, causing epigenetic silencing of the *MSH2* gene promotor [30,40,41,42,43]. Rare exceptions exist and are described later.

### 3.2. Lynch Syndrome Versus HNPCC

Nowadays, the term LS is still frequently used interchangeable with Hereditary Nonpolyposis Colorectal Cancer (HNPCC), a term introduced in 1984 [30]. However, this usage is not entirely accurate for three reasons.

First, LS patients can present with one to a few polyps [14,15]. Second, HNPCC refers to patients who meet the Amsterdam criteria (presented in a later section), and this includes not only individuals with LS, but also those with Constitutional Mismatch Repair Deficiency (CMMRD—a recessively inherited disorder involving the same genes affected in LS) or Familial Colorectal Cancer Type X (FCCTX—a heterogeneous HCRCS characterized by a cancer pattern similar to LS but lacking MSI, with responsible genes remaining unknown or controversial, including: *APC*, *BMPR1A*, *BRCA2*, *BRF1*, *FAF1*, *FAN1*, *GALNT12*, *NTS*, *OGG1*, *RASSF9*, *RSP20*, *SEMAN4A* and *SETD6*, among others) [44,45,46,47]. Third, the term “Lynch syndrome” more accurately reflects the fact that beside CRC, pathogenic variants in MMR genes confer an increased risk for a broad spectrum of cancers, including endometrium, ovaries, and stomach cancers [10].

For these reasons, LS is the preferred designation for the cancer predisposition syndrome caused by heterozygous variants in MMR genes, while HNPCC is a limited description of this syndrome and should be avoided.

### 3.3. LS Phenotypic Variability and Related Phenotypes

An increasingly recognized group of CRC cases is classified as Lynch-like syndrome (LLS). By definition, LLS refers to CRC cases that exhibit MSI with loss of expression of one or more MMR proteins, but in which neither (likely) pathogenic variants could be identified in the corresponding MMR genes (*MLH1*, *MSH2*, *MSH6*, *PMS2*, or *EPCAM*), nor *MLH1* promoter methylation or the somatic *BRAF* V600E mutation [45].

Although these cases could be sporadic tumours with somatic biallelic inactivation of MMR genes, they likely represent undetected HCRCS or LS cases, due to the nature of the underlying variants or technical limitations of currently applied molecular screening methods [45,46].

There are phenotypes or clinical variations in LS.

The Muir–Torre syndrome (OMIM 158320), independently described in 1967 by Muir et al. [47] and in 1968 by Torre [48], is characterized by the presence of benign and malign, mostly sebaceous skin tumours, in combination with CRC and other malignancies [49]. The most frequently affected gene is *MSH2* in 90% of the Muir–Torre cases, although pathogenic variants in *MLH1*, *MSH6* and *PMS2* were occasionally reported [50,51,52].

The Turcot syndrome was described in 1959 [53] and is characterized by brain tumours such as glioblastoma multiforme, occurring alongside CRC or adenomas [41,49]. It was later proposed dividing Turcot syndrome into two subtypes: Turcot Syndrome type I with MMR pathogenic variants, whereas Type II shows *APC* pathogenic variants.

Finally, Constitutional Mismatch Repair Deficiency (CMMRD—a recessively inherited disorder (OMIM #276300)) is also caused by pathogenic variants in MMR genes. This condition is very rare and involves high-grade gliomas, T-cell lymphoblastic lymphoma, CRC and other LS-associated cancers [54], and only around 200 have been reported worldwide [14,55]. CMMRD among all syndromes confers the highest cancer risk, developing during the first year of life, with half of the patients developing cancer before the age of ten, and 80% to 90% before the age of 18 [54,56].

The first case of CMMRD was presumably described by Wang et al. who identified a constitutional homozygous inactivation of the *MLH1* gene in a child with clinical features of de novo neurofibromatosis type 1 and early-onset extracolonic cancers [57]. Regarding the spectrum of pathogenic variants, *PMS2* stands out with 60%, followed by *MSH6* (>20%), and *MLH1* and *MSH2* (together <20%). The observed lower penetrance of monoallelic *PMS2* and *MSH6* alterations might escape LS diagnosis and thus explain why their incidence is higher among CMMRD patients [54]. This gene-dependent penetrance is further reflected by the overall patient survival at age 15: 63% for *PMS2*, 49% for *MSH6*, 19% for *MLH1*, and 0% for *MSH2*) [56].

## 4. Molecular Genetics of Lynch Syndrome

### 4.1. DNA Mismatch Repair

*MLH1*, *MSH2*, *MSH6* and *PMS2* constitute the LS-relevant core group of MMR genes (and respective proteins). They are highly conserved across species and play a key role in post-replicative DNA repair to maintain genomic stability [58]. Related genes are *PMS1*, *MLH3*, *MSH3*, *MSH4* and *MSH5*; however, no conclusive evidence links pathogenic variants in *MSH3* and *MLH3* to LS, and *PMS1*, *MSH4*, and *MSH5* are involved in meiotic recombination rather than MMR [14]. In LS, *MLH1* and *MSH2* are the most frequently mutated, as they are essential for the proper functioning of the DNA repair complex, followed by *MSH6* and *PMS2* [14,59,60].

**Figure 2:**
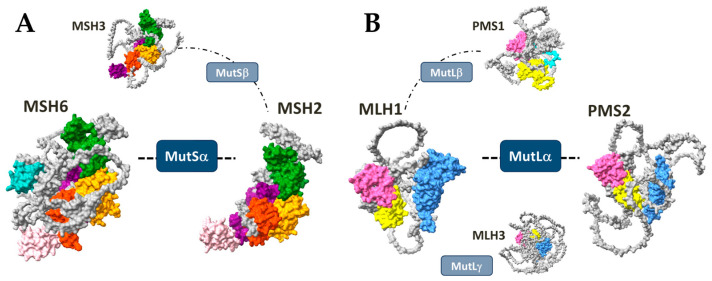
Protein structure models of heterodimers formed by human MSH2 and MLH1. The predicted 3D models visualize the structural similarities among the MutS complex partners MSH2, MSH3, and MSH6 (**A**), or the MutL complex partners MLH1, PMS2, MLH3, and PMS1 (**B**). The colour-coded areas (see Supplementary Legend S1 for a more detailed description) demonstrate the structural and functional conservation of protein domains among paralogs in which most of the pathogenic variants are known to occur. The larger size of the MSH6, MSH2, PMS2 and MLH1 images reflect their relevance in LS.

When DNA polymerases fail in their proofreading activity, a heterodimer of MSH2 and MSH6 proteins, called the MutSα complex, recognizes single-base pair mismatches or misaligned loops caused by 1–2 insertions or deletions, but also larger loops (up to 8–10 nucleotides) [14]. In contrast, MSH2-MSH3 heterodimers, known as the MutSβ complex, recognize only larger loops (Figure 2). These heterodimers can discriminate between the newly synthesized and the parental DNA strand that serves as template for DNA sequence correction [41,61].

When one of the two MutS complexes has recognized a mismatch, it recruits under ATP hydrolysis MLH1 with one of its heterodimeric binding partners: PMS2 (MutLα complex), PMS1 (MutLβ complex), or MLH3 (MutLγ) [41,58,62]. Together, they form a tetrameric complex (MutS and MutL) that moves like a sliding clamp along the DNA until it encounters a single-stranded discontinuity. At this point, the newly synthesized DNA strand requiring correction is identified and excised by the complex-associated exoncuclease EXO1. The replacement process is then carried out through the coordinated activity of several proteins, including DNA polymerase δ (Polδ)—which incorporates the correct nucleotides, PCNA—which stabilizes Polδ and helps in the recruitments of MutL complex and EXO1, and DNA Ligase I—which religates the newly synthesized DNA strand. Other proteins, such as Replication Protein A (RPA), function as stabilizers and signalling molecules, flagging the damage site and recruiting Replication Factor C (RFC), and also contribute to this highly complex process [63,64].

As part of the cellular response to DNA damage, the MMR pathway also plays a role in apoptosis induction and cell cycle arrest, in case the damage cannot be repaired. In particular, MMR proteins are required for activation of the DNA damage-associated protein kinases ATM and ATR, eventually leading to activation of the p53 signalling pathway. It is still unknown whether this occurs due to futile DNA repair cycles leading to double-strand breaks, or to direct signalling of MMR proteins to ATM/ATR [41].

Recently, the helicase activity of homologous recombination factor Minichromosome Maintenance protein 9 (MCM9) was found to be required for MMR and involved in the MSH2-dependent recruitment of MLH1 [65,66]. If confirmed, a link between the Homologous Recombination Repair (HRR) and MMR pathways could lead to new clinical and therapeutic opportunities [67].

### 4.2. Prevalence of Pathogenic LS Variants in MMR Genes

In most LS cases, patients inherit pathogenic variants in the *MLH1* or *MSH2* genes from one of their parents. Only approximately 2.3% represent de novo variants, as suggested by Win et al. by analyzing 261 probands [68]. The predicted prevalence of LS varies across studies, ranging from approximately 1/250 to 1/1000, depending on the size and origin of the populations analyzed [69]. According to data from the Prospective Lynch Syndrome Database (PLSD) that registered pathogenic LS variants in any of the MMR genes across 25 countries and 8500 carriers (4588 females and 3912 males), *MLH1* (36.8%) and *MSH2* (37.3%) were the most frequently affected genes, followed by *MSH6* (19.4%) and *PMS2* (6.2%) [70]. These findings are consistent with those of Thompson et al., who analyzed 2360 unique MMR gene variants collected years earlier from the InSiGHT locus-specific database, with only minor differences: *MLH1* was the most frequent (39%), followed by *MSH2* (36%), *MSH6* (19%) and *PMS2* (6%) [60]. Some LS patients may also have more than one MMR affected gene that is known as digenic LS [41].

Interestingly, Win et al. observed that the prevalence of gene variants is higher for *PMS2* (1/714) and *MSH6* (1/758) when compared to *MLH1* (1/1946) and *MSH2* (1/2841), although LS is less frequently diagnosed in carriers of *PMS2* and *MSH6* variants [71]. This lower penetrance of *PMS2* and *MSH6* genes is also responsible for the rare CMMRD cases discussed above. A higher LS penetrance (1/226) compared to other populations characterizes the Icelandic population because of the presence of founder variants in *PMS2* and *MSH6* [72].

Mechanistically, the lower penetrance of *PMS2* variants is believed to be related to the ability of MLH3 and PMS1 proteins to compensate for non-functional PMS2 by forming heterodimers with MLH1 [73]. A similar mechanism may occur for *MSH6* variants due to the alternative complexation of MSH2 with MSH3.

### 4.3. Cumulative Cancer Risk

Although carriers of pathogenic variants that affect MMR genes were thought to share a similar cancer risk, primarily for CRC and EC, both retrospective and prospective observational studies [59,74,75] revealed that this risk depends not only on the penetrance of the affected gene, but also on the type of variant, resulting in distinct phenotypic expression of LS.

According to data from the PLSD, germline pathogenic variants in *MLH1* and *MSH2* are primarily linked to an increased risk of CRC and EC and are classified as high-penetrance genes. The cumulative cancer risk ranges from 64% to 78% by age 70 [59,74]. Pathogenic variants in *MSH6* and particularly in *PMS2* are most strongly associated with EC. Here, the cumulative cancer risk for *MSH6* is 62% in women by age 70, compared to 28% in men, and for *PMS2* 22% by age 70 [14,70].

Concerning the cancer spectrum of LS, a prospective follow-up of 1436 patients revealed colon cancer in 481 cases (26%), followed by 237 EC (12.8%), 155 skin cancers (8.4%) and 137 rectal cancer (7.4%) [76]. Depending on the inherited MMR variant, LS also increases the risk of ovarian, stomach, small bowel, bile duct, pancreatic, and upper urinary tract cancers [74,77].

### 4.4. Is There Room for More in LS Classification?

In 2023, Møller et al. proposed that Lynch syndrome (LS) should be subdivided into four distinct types, as it is caused by pathogenic variants in four different genes (*MLH1*, *PMS2*, *MSH6*, and *MSH2* or 3′ *EPCAM* deletions that result in *MSH2* silencing), each with specific clinical features and outcomes. For example, pathogenic *MSH6* variants are associated with mostly EC development at older ages in females, while *MLH1* variants confer higher risk of CRC, gastric and pancreatic cancers in male than in female *MLH1* carriers [78].

The authors proposed naming the different LS subtypes after the affected gene (e.g., OMIM #120435 for *MSH2*, OMIM #609310 for *MLH1*, OMIM #614350 for *MSH6*, OMIM #614337 for *PMS2*, OMIM #613244 for *EPCAM* deletion [78,79]), and this would simplify the future nomenclature in case novel genes causing MSI cancers will get identified, considering the rapid and continuous advancement of sequencing technologies, methodologies, and diagnostic strategies. Such genes may also encompass non-coding elements, with microRNAs (miRNAs) at the forefront that can downregulate MLH1 (miRNA-155) or MSH2 and MSH6 (miRNA-21) protein levels [45,80].

### 4.5. Somatic Event—The Second Hit

Although the gene variant penetrance among LS patients does not reach 100% [14], carriers have a significantly higher cumulative risk of developing cancer compared to the general population [81]. Like all tumour suppressor genes, the complete loss of MMR function occurs following Knudson’s two-hit model, in which the second allele suffers a somatic event that inactivates the respective gene in a cell [6,41,82,83]. Currently, it is well established that the “second hit” and subsequent inactivation of the wild-type allele can also occur through epigenetic mechanisms, such as *MLH1* promoter methylation, rather than through the acquisition of a de novo pathogenic variant in the nucleotide sequence [84]. It should be noted that haploinsufficiency was observed in heterozygous *mlh1* and *msh2* transgenic mice [85,86], which could further accelerate tumour development.

After the ‘second hit’ has occurred, both MMR alleles are non-functional, leading to deficient MMR (dMMR). This results during each DNA replication cycle in the accumulation of somatic variants in repetitive sequences present in microsatellites, but also in coding genes and genomic regions. This mutator phenotype generates a 100 to 1000-fold increase in the mutation rate [87], ultimately promoting cell growth dysregulation, reduced apoptosis response, and oncogenesis. Consequently, the progression from adenoma to CRC in LS patients occurs rapidly within 2 to 3 years, particularly in carriers of pathogenic variants in *MLH1* or *MSH2* genes [4,88].

### 4.6. Molecular Pathways of Oncogenesis in LS

Analysis of non-tumorous mucosa of LS patients identified MMR-deficient colon crypt foci (MMR-DCF), which exhibit a histologically normal appearance, and occur as one MMR-DCF per cm^2^ of non-tumorous mucosa. Despite their high abundance in LS patients, only a subset of these lesions is believed to progress to adenomas or carcinomas [73,89]. One possible explanation is the replacement of dMMR stem cells in the colonic mucosa by mismatch repair-proficient MMR (pMMR) stem cells within affected crypts [89,90]. In addition, dMMR alone is insufficient for adenoma formation and depends on the acquisition of other oncogenic alterations [73,89,91].

The identification of MMR-DCF allowed to obtain important insights into how CRC can develop in LS patients [89] and indicated three distinct pathways. In pathway 1, CRC develops from pMMR adenomas after *APC* loss of function (LoF) and is most common in *PMS2* and *MSH6* pathogenic variant carriers. Pathway 2 is believed to evolve from morphologically normal MMR-DCF, and is predominantly associated with *MSH2* LoF, followed by *APC* inactivation with subsequent adenoma formation, following the conventional adenoma-carcinoma sequence [91]. Pathway 3-associated CRC cases also progress from MMR-DCF but as flat lesions of which approximately 50% harbour somatic *CTNNB1* variants, show frequent *TP53* LoF and a rapid invasive growth pattern [73,91,92]. In one study, 17.4% of LS tumours harboured pathogenic *CTNNB1* variants and developed as intramucosal flat lesions with histological features suggesting immediate invasive growth [93], undetectable by regular colonoscopic surveillance.

After reviewing 640 CRC cases in LS patients, Ahadova et al. further elucidated that flat tumours, believed to originate from MMR-DCF, may either exhibit secondary *APC* inactivation (Pathway 2), or pathogenic *CTNNB1* variants and *TP53* inactivation (Pathway 3). Only 23.3% of adenomas in LS exhibit pMMR (Pathway 1) [91].

Interestingly, the presence or absence of adenomas appears to be associated with the specific MMR gene affected by the germline variant. Indeed, Engel et al. observed that among 2747 LS patients, adenomas were significantly more common in *MSH2* and *MSH6* pathogenic variant carriers than in *MLH1* carriers. Additionally, the risk of advanced adenomas was higher in *MSH2* pathogenic variant carriers than in *MSH6* and *MLH1* carriers. The same study observed a higher incidence of CRC in *MLH1* pathogenic variant carriers, which indicates that these tumours often arise from flat lesions with *CTNNB1* variants (Pathway 3), a process that may be associated with a more rapid progression to malignancy. Meanwhile, tumours from *MSH2* pathogenic variant carriers more frequently exhibit secondary *APC* variants compared to those from *MLH1* carriers, which may explain the higher incidence of adenomas among *MSH2* carriers (Pathway 2) [75,92].

The lower incidence of CRC among *MSH6* and *PMS2* pathogenic variant carriers also aligns with the described pathways, because adenomas in *MSH6* and *PMS2* carriers exhibited a lower frequency of dMMR compared to other LS carriers, but a higher frequency of pathogenic *APC* variants, suggesting that dMMR likely occurs after adenoma development through Pathway 1. *PMS2* mutant adenomas furthermore show unaffected *CTNNB1.* This may explain why *MSH6* and especially *PMS2* pathogenic variant carriers have a lower risk of CRC [73,75,94].

*CTNNB1* encodes β-catenin, a protein involved in cell adhesion and the regulation of cell growth through the Wnt pathway. In the absence of Wnt ligand, a cytoplasmic protein complex composed of APC, Axin-1, Axin-2, and β-TrCP, promotes β-catenin phosphorylation and subsequent proteolytic degradation. This prevents its accumulation and translocation to the nucleus where it acts as a coactivator of transcription factors from the T-cell factor/lymphoid enhancer factor (TCF/LEF family), ultimately activating Wnt-responsive genes [95,96,97]. Thus, tumours harbouring activating *CTNNB1* variants do not require *APC* inactivation [98].

Although the enrichment of *CTNNB1* variants in LS cases is not fully understood, this phenomenon may be explained by the genomic proximity of *CTNNB1* and *MLH1*, both located less than 5 Mbp apart on chromosome 3p22.1-p22.2. This proximity could account for the frequent occurrence of somatic *CTNNB1* variants in *MLH1* carriers (47% in a recent study) [92]. According to this hypothesis, termed the “2-in-1 hit,” both alleles may co-segregate simultaneously through the mitotic recombination event of copy number-neutral loss of heterozygosity (cnLOH). Notably, the authors demonstrated cnLOH in all 5 tumours that were screened by whole exome sequencing (WES). Additionally, 16 of 21 samples (with *CTNNB1* variants) also presented several SNPs in *CTNNB1* with variant allele frequencies above 80%, consistent with homozygosity resulting from cnLOH. Interestingly, all 21 samples exhibited variants affecting the typical oncogenic codons 41 or 45 (c.121A>G (p.Thr41Ala), and c.133T>C (p.Ser45Pro) located in exon 3 of *CTNNB1*) [92]. Indeed, it is well established that Ser45 (S45) is phosphorylated by Casein Kinase I isoform alpha (CSNK1A1) at the N-terminus, which then allows Glycogen Synthase Kinase-3 (GSK3) to phosphorylate Tyr41 (T41), Ser33 (S33) and Ser37 (S37). It is well documented that residues 32 to 37 (DSGIHS) correspond to the β-TrCP consensus binding motif (DSGXXS), which is essential for ubiquitin conjugation that marks β-catenin for degradation through the proteasome [97,98,99].

## 5. Guidelines for Molecular Screening and Clinical Management of LS

Henry Lynch originally reported an average age of 45 years at diagnosis, substantially earlier than sporadic CRC in the general population with an average age of 69 years [100,101]. Meanwhile, however, the estimated age of onset LS-associated cancers is known to depend on the gene and the specific pathogenic variant involved. The PLSD allows for the estimation of the respective cumulative risk for various tumour types, including CRC, stratified by age and sex [70]. Analysis of data in the PLSD also indicated that colonoscopic surveillance in LS patients did not reduce CRC incidence, unlike sporadic CRC cases, based on reports that patients developed CRC within three years of a previously negative colonoscopy [16]. This suggests that these tumours likely evolve rapidly (possibly between colonoscopies) and/or were difficult to detect during colonoscopic examinations [41].

### 5.1. Guidelines to Identify LS Patients and Families for Genetic Testing

The Amsterdam Criteria (I) were the first guidelines developed in 1990 for the identification of LS patients [102]. Since it became evident that these criteria identified only about 60% of LS cases, they were revised in 1999 in the Amsterdam II Criteria [103] in order to include other LS-associated cancers, which improved sensitivity for LS detection to approximately 80% [101,103,104,105].

Additionally, Bethesda Guidelines were developed independently in 1996 by the National Cancer Institute (NCI) in Bethesda and published in 1997. The primary aim was to establish clinicopathologic criteria to identify additional LS patients who were not detected using the Amsterdam Criteria alone [106]. In 2004, the Bethesda Guidelines were also revised [107,108].

Today, both the revised Bethesda Guidelines and the Amsterdam II Criteria continue to be widely used to identify individuals with LS, as reflected in the ACMG Guidelines for Genetic Testing for Inherited CRC and Polyposis (2021 revision), as well as in the most recent guidelines of the National Comprehensive Cancer Network (NCCN) (Version 1.2025) [99,101]. Meanwhile, NCCN recommends that all cases diagnosed with CRC or EC are screened for their MMR status by IHC. This has substantially improved the identification of individuals at risk for LS compared to clinical criteria alone [13,109]. In addition, patients with dMMR tumours may benefit from immune-checkpoint inhibitor therapy, an effective precision medicine approach [110].

In LS patients, approximately 70% to 80% of tumours arise in the proximal to transversal colon—substantially more frequent than the 30% observed in sporadic CRC cases. This anatomical characteristic may serve as a clinical indicator of LS suspicion in individuals with unknown germline variant status.

### 5.2. Molecular Diagnosis of LS

A definitive diagnosis of LS requires genetic testing in index patients [111]. Three molecular screening layers are used in routine clinical practice.

(a) Microsatellite Instability evaluation

There are two distinct methods, either by immunohistochemistry (IHC) of MMR proteins or by MSI testing via PCR, both with high sensitivity and specificity, though neither is 100% accurate [14,99,112,113]. The evaluation of dMMR by IHC detects the presence or absence of nuclear staining in tumour cells using antibodies against the four MMR proteins—MSH2, MSH6, MLH1, and PMS2—with internal positive control signals being provided by adjacent non-neoplastic cells [14,111]. Lack of IHC staining sometimes offers the advantage of hinting at the affected genes.

MSI testing by PCR evaluates loci such as those described and recommended by the Bethesda panel, by the National Cancer Institute in 1997, which includes two mononucleotide “Big Adenine Tract” loci (BAT-25 and BAT-26) and three dinucleotide loci (D2S123, D5S346, and D17S250) [114], comparing tumour and matched normal DNA. Tumours exhibiting instability in two or more loci are classified as MSI-high (MSI-H) while those showing no instability in any of the five loci are considered microsatellite stable (MSS). Cases with instability in only one locus are categorized as MSI-low (MSI-L) [115].

Pearlman et al. concluded that dMMR/MSI detection using IHC and PCR provides comparable negative predictive values (99.6%) and shows 99.1% concordance between methods. IHC demonstrated a sensitivity of 88.9%, which was lower than that of PCR-based MSI detection (92.9%), while exhibiting slightly higher specificity (87.9% vs. 86.3%) [116]. However, in clinical settings, MSI testing is less frequently performed due to the small size of the tumours, with a higher rate of non-performance compared to IHC (14% versus 0.3%, respectively) [45,99].

With the advent of next-generation sequencing (NGS), gene panel screening and MSI analysis of homopolymer indels or microsatellite length variability, the sensitivity and specificity values achieve 95% and 98%, respectively, as well as a 97% concordance with PCR and IHC, according to Trabucco et al. [64,117,118,119].

The currently published NCCN Guidelines recommend the use of one or both methodologies [64,99,120], primarily due to the poor performance of the Bethesda guidelines in detecting carriers of the *MSH6* pathogenic variants, and to a lesser extent, of the *PMS2* and *MSH2* genes [13]. Similarly, the recommendation to test all EC cases was proposed due to the low specificity of the Bethesda and Amsterdam II criteria in identifying LS patients, with specificities of 49% and 61%, respectively [113,121].

(b) BRAF V600E and *MLH1* promotor hypermethylation

In cases where routine analysis of a CRC sample exhibits loss of *MLH1* expression by IHC, it is recommended to screen for somatic *BRAF* V600E mutation and *MLH1* promoter methylation in a tumour sample, prior to conducting NGS panel testing for MMR genes [109,120]. This is because both *BRAF* V600E mutation and *MLH1* promoter methylation are generally only observed in sporadic CRC cases [122]. It should, however, be noted that approximately 1.5% of tumours from LS patients may also harbour somatic *BRAF* V600E mutation, and *MLH1* promoter inactivation may serve as the second hit in LS individuals carrying pathogenic *MLH1* variants [123,124,125]. Although very rare, germline *MLH1* promoter hypermethylation has also been documented [116,126].

Technically, evaluation of the somatic *BRAF* V600E mutation in tumour DNA is commonly performed using restriction enzyme digestion, allele-specific primer extension or real-time PCR [9]. Analysis of *MLH1* promoter region “C”—a small proximal segment (NM_000249.4: c.−248 to c.−178) where hypermethylation status consistently correlates with *MLH1* gene silencing—is typically conducted using bisulfite modification followed by real-time quantitative methylation-specific PCR (qMSP) to detect both methylated and unmethylated alleles [9,127]. An alternative methodology is methylation-specific multiplex ligation-dependent probe amplification (MS-MLPA) that enables the simultaneous detection of the *BRAF* V600E mutation and *MLH1* promoter methylation.

(c) Multigene Panel Testing (NGS), MLPA and Sanger sequencing

The application of NGS methodologies for the simultaneous screening of multiple genes, either through capture hybridization or amplicon-based target enrichment, has significantly reduced turnaround time for genetic screening in LS patients, which was previously conducted using Sanger sequencing and multiplex ligation-dependent probe amplification (MLPA) [9,128]. Often referred to as multigene panel testing (MGPT), this method should include the analysis of coding exons, intron/exon boundaries, promotor regions and relevant regions of 5′ and 3′ UTR of *MLH1*, *MSH2*, *MSH6*, *PMS2*, and *EPCAM*, with the evaluation of *EPCAM* focusing only on large deletions in the 3′ region [99,109,120,128].

Although several bioinformatic tools for in silico CNV detection, have been developed to analyze NGS data, MLPA is still widely used in good practice for confirming CNVs identified by NGS due to the considerable number of false positives generated by computational algorithms [129,130,131,132]. However, MLPA is limited to the specific regions where probes hybridize, and in silico analysis may detect a greater number of CNVs [101,128]. For instance, in the study by Moreno-Cabrera et al., the use of the DECoN tool on 2041 samples resulted in 158 CNV calls, of which only 19 were confirmed as true CNVs. On the other hand, the authors also identified eight CNVs that were missed by MLPA due to its restricted probe coverage [130].

The traditional Sanger sequencing is still useful in the workflow analysis of LS, either to confirm variants detected by NGS or sequence regions not covered adequately by NGS, and in rapid predictive testing of asymptomatic family members [9,101,128] following the identification of the LS-causing variant in index cases [9,14,120,133].

### 5.3. MMR Gene Variant Classification Guidelines

Since 2011, Expert Panels from the International Society for Gastrointestinal Hereditary Tumours (InSiGHT) and the American College of Medical Genetics and Genomics (ACMG) and the Association for Molecular Pathology (AMP) developed 5-tiered schemes for a standardized variant classification [60,133], later transformed from probabilistic boundaries into a quantitative Bayesian framework [134]. In this Bayesian framework, pathogenicity evidence points are assigned as follows: Very Strong = 8, Strong = 4, Moderate = 2, Supporting = 1. Conversely, benign criteria are scored as Very strong = −8, Strong = −4, Moderate = −2, Supporting = −1. The cumulative score, depending on the applied criteria, determines the final classification of the variant according to the following thresholds: ≥10 (Pathogenic), 6–9 (Likely Pathogenic), 0–5 (Variant of uncertain significance (VUS)), −1 to −5 (Likely Benign), ≤−6 (Benign).

In 2024, the InSiGHT MMR guidelines were updated in collaboration with ClinGen [135], with 18 of the original 28 ACMG/AMP criteria considered applicable. In the same year, CanVIG-UK released its MMR guidelines, which showed significant differences in both the number of criteria applied and the scoring weights assigned to 12 criteria [136].

Meanwhile, computational tools, the original ACMG criteria and the scoring weights attributed to certain criteria were harmonized between panels in 2025, with the latest version of the CanVIG-UK guidelines (v3.21) published on 4 February 2026 [135,137].

Intragenic CNVs (deletions or duplications above 50 pb) [138], should be classified with the specific MMR guidelines; however, the interpretation of CNVs that affect several genes have specific guidelines [139,140].

### 5.4. Prevalence and Type of Mismatch Repair Gene Variants

The worldwide recognized MMR variant database is maintained by InSiGHT, sharing data with both the Leiden Open Variation Database (LOVD) and the ClinVar database, and contains a total of 8027 MSH2, 6168 MLH1, 9997 MSH6 and 5691 PMS2 germline variants. Among these, a substantial proportion are classified as VUS, with the majority presenting as missense variants (1757 in *MSH2*, 1718 in *MLH1*, 3863 in *MSH6* and 2448 in *PMS2* [141,142].

The number of variants with a consolidated three-star expert review status is currently 1714 variants [41,141,142,143]. Among these, 41% were identified in *MLH1* (702 variants), followed by 37% in *MSH2* (642 variants), 15% in *MSH6* (263 variants) and 6% in *PMS2* (107 variants). When considering only pathogenic and likely pathogenic variants, the proportions remain similar: *MLH1* accounts for 43% (597 variants), *MSH2* for 39% (544 variants), *MSH6* for 13% (184 variants) and *PMS2* for 5% (69 variants). At the molecular level, these variants encompass predominantly frameshift variants (36%), followed by nonsense (20%), splicing (15%) and missense (13%) variants. Splice site variants at acceptor and donor sites (8% and 7%, respectively) are defined as changes in the last three exonic to the first six intronic bases at the acceptor, and the last 12 intronic to the first two exonic nucleotides at the donor site. These can alter the strength of native splice sites (including branch point sites) or even create novel splice sites [144,145,146]. Such effects can be predicted using in silico tools, such as SpliceAI [147], as recommended by the InSiGHT ClinGen and CanVIG-UK MMR variant classification guidelines, and when necessary predictions should be functionally characterized (e.g., SpliceAI ≥ 0.2) [135,136,148].

In addition, among the reviewed missense variants, approximately 60% actually disrupt splicing [41] and should therefore be considered truncating variants. Thus, the identification of a missense variant in LS poses a double challenge: most of them are classified as VUS, but may also require assessment for potential effects on splicing. In light of the above harmonized guidelines [137] some variants currently listed in ClinVar may be subject to reclassification [135]. In case the final classification remains as VUS, further clinical management of the patient is generally phenotype-driven, based on tumour phenotype, family history, age at onset, etc.

Large rearrangements are also notable, accounting for 12% of expert-reviewed cases. Notably, 32% of pathogenic variants in *PMS2* (22 variants) are due to large deletions, a frequency considerably higher than observed in other MMR genes, namely 17% in *MSH2* (92 variants), 8% in *MLH1* (48 variants) and 4% in *MSH6* (8 variants). Nevertheless, a higher absolut number of deletions in *MSH2* and *MLH1* exist compared to other MMR genes, and breakpoints are frequently located within *Alu* repeat regions [40], in which the *MSH2* genomic region is particularly enriched. Interestingly, long interspersed nuclear elements (LINEs), which are abundant in *MLH1*, do not appear to significantly contribute to the formation of deletions, as they are less recombinogenic than *Alu* elements [40].

Duplications are less frequently reported, representing only 0.5% (9 variants); however, this may partially reflect the known limitations of in silico tools in reliably detecting duplications.

### 5.5. Founder Variants

Some variants are considered as founder events that appeared centuries ago in genetically isolated populations, including more than 40 single nucleotide variants in *MLH1, MSH2* and *MSH6*, a large duplication in *MLH1* and several deletions (4 in *MLH1*, 6 in *MSH2*, 1 in *MSH6* and 2 additional deletions in exons 8 and 9 of *EPCAM*) [149].

Regarding *PMS2* founder variants, Tomsic et al. reported more than 10 variants observed in unrelated individuals, including two large deletions (exons 5–7 and exon 10) [150]. Additional founder variants were identified in the Icelandic and Norwegian populations, contributing new variants to the list reported by [72,151].

## 6. Functional Assays to Classify VUS in Mismatch Repair Genes

**Figure 3:**
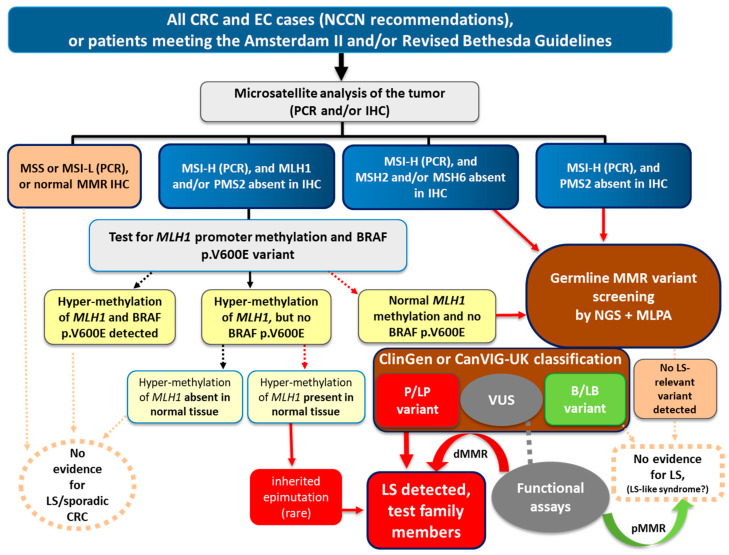
Schematic workflow for variant diagnosis and classification. The scheme illustrates how the status of microsatellite instability, immunohistochemistry, *MLH1* promoter methylation, and *BRAF* genotype select samples for germline testing, followed by InSiGHT ClinGen and CanVIG variant classification and, in case of VUS, by assays to test the functional effect of missense variants in MMR gene.

For an effective MMR pathway, the involved proteins first need to be transcribed, generate stable transcripts and be translated. In case of missense variants, the respective MMR protein must be able to form the appropriate heterodimers, and ultimately support DNA repair or DNA damage signalling in the nucleus. A failure at any of these stages will result in dMMR, MSI, and survival of damaged cells. Unfortunately, a considerable number of the identified missense variants are VUS that do not allow per se appropriate classification and thus clinical management of patients and healthy carriers. To this end, the resulting variant proteins require functional assays to characterize and understand their biological impact. Considering the shift in molecular diagnosis to NGS and the availability of functional assays, Figure 3 presents an updated workflow scheme for variant diagnosis and classification. Depending on the type and design of the functional assay, variant classification guidelines such as ClinGen and CanVIG-UK attribute different scores or strengths to different types of assays.

**Figure 4:**
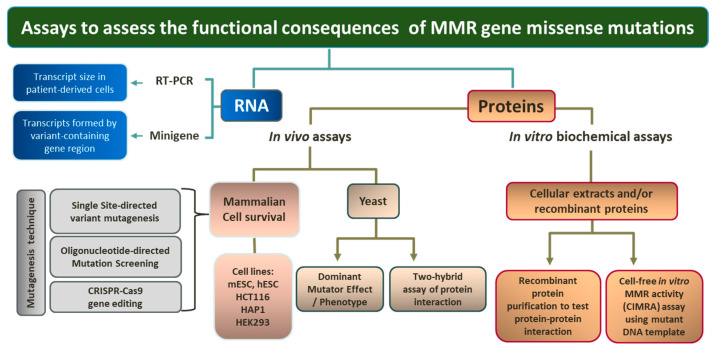
Schematic overview of the different published strategies employed to test the functional effect of missense variants in MMR genes.

Functional assays can be broadly divided into two main categories (Figure 4): those that study the effect of variants at the messenger RNA level (which are well documented elsewhere and will only be briefly described here), and those aimed at assessing the impact at the protein level, either in vitro or in vivo.

### 6.1. Variant Effect at Messenger RNA Level

To determine a variant’s effect at the splicing levels, RNA can be extracted from patient blood cells or other tissues. Then, reverse transcription PCR (RT-PCR) allows for the examination of specific transcript regions or the full-length transcript [144,145,152,153]. This allows the evaluation of exon skipping, cryptic splice site usage or intron retention to confirm in silico predictions (e.g., SpliceAI) [148,154].

Alternatively, when patient RNA cannot be obtained, the identified variant sequence can be subcloned into a minigene construct and then transfected into cultured human cells. This approach was thoroughly described by Cooper et al. and Gaildart et al., and can serve as a complement to patient mRNA analyses [152,155].

Briefly, an exon sequence containing the genetic variant of interest is PCR-amplified together with approximately 150 bp of flanking intronic regions from patient genomic DNA. The oligonucleotide primers used for this amplification include restriction enzyme recognition sites (e.g., BamHI and MluI) that are also present in the cloning vector. Then, the amplified fragment is inserted into an intron of a minigene vector between two constitutively spliced exons. The resulting minigene is then transfected into human cells where it is transcribed under the control of a promotor. Then, total RNA is extracted from cells followed by RT-PCR amplification of the minigene-derived transcripts. RT-PCR products are subjected to electrophoresis to visualize whether the variant-containing exon was included. All bands can then be gel-purified and sequenced to determine the splicing pattern of transcripts derived from wild-type and variant constructs [152,155,156]. Minigene constructs have been very useful but may not always reflect the endogenous splicing regulatory context [152].

### 6.2. Variant Effect at Protein Level

Broadly, assays that evaluate the impact of missense variants at the protein level can be categorized as in vitro or in vivo. In vitro assays have certain limitations, as they use cellular extracts or recombinant proteins, and do not replicate the entire native cellular environment, including chromatin context and potentially unknown cofactors or interacting protein partners. In vivo assays, on the other hand, provide these cellular factors in either yeast, murine or human embryonic stem cells (mESCs/hESCs), or other human cell lines.

#### 6.2.1. Cell-Free In Vitro MMR Activity (CIMRA) Assay

Early studies investigated the effect of variants on the direct protein–protein interaction of recombinant MLH1 and PMS2 using glutathione S-transferase (GST)-pull down assays [157]. Later, the CIMRA assay developed by Drost et al. tested repair activity by incubating an engineered plasmid-based mismatch repair substrate with purified recombinant MMR proteins, either wild-type or variant [158,159].

The substrate plasmid, initially derived from pUC19CD, contains a G–T mismatch within a HindIII restriction site and a fluorescent label (6-FAM), resulting in the construct pJHGT3lnFAM. Recombinant MMR proteins are first cloned by inserting the cDNA into the pCITE4a plasmid as a template. After site-directed mutagenesis (s-dm), to introduce the desired variants, the constructs are subjected to in vitro transcription and translation to synthesize the recombinant proteins [159,160].

Each recombinant protein is incubated for 15 min at room temperature with its corresponding wild-type dimerization partner, also expressed in vitro from pCITE4a, to allow heterodimer formation. These recombinant dimers are then incubated for 40 min at 37 °C with the mismatch-containing plasmid (pJHGT3lnFAM) and nuclear extracts from either LoVo or HCT116 cells that each lack the expression of endogenous MSH2/MSH6 or PMS2/MLH1 proteins, respectively [159,160].

Following incubation, DNA is extracted and digested with HindIII. If the variant is functional, the G–T mismatch is repaired, which restores the HindIII recognition site, yielding a 75 bp fluorescent fragment that is detected and quantified by capillary electrophoresis. In contrast, if the variant is functionally defective, the mismatch is not corrected, and the restriction site remains disrupted, preventing cleavage [159,160]. This assay has been successfully applied by Drost et al. for the functional characterization of several *MLH1*, *MSH2*, *MSH6* and *PMS2* variants in a series of publications [158,159,160,161,162,163].

A similar approach to the CIMRA assay has been developed by the Nyström group and Geng et al. They employed circular DNA substrates containing either a mismatched base pair or a small insertion at an endonuclease recognition site. However, in these studies the test variants were overexpressed in insect cells (Sf9 and High Five) and purified, rather than being produced as above via in vitro protein translation [164,165,166,167].

More recently, González-Acosta et al. or Takahashi et al. described in vitro MMR assays, which also use a circular DNA substrate, but MMR proteins were purified from human embryonic kidney 293T (HEK293T) or colorectal HCT116 cells [168,169], after their transient transfection with plasmids carrying the variants of interest.

Compared to the CIMRA, these approaches offer the key advantages that the nuclear extracts contain the remaining MMR machinery, and that differences in the translation efficiency of the variant protein in a human cell can be determined by normalization to a co-transfected GFP control protein [169].

#### 6.2.2. Yeast In Vivo Assays

The yeast *Saccharomyces* (*S.*) *cerevisiae* has been widely used as a model organism in functional assays due to the evolutionary conservation of the MMR pathway between yeast and human cells. Yeast-based assays can evaluate parameters such as forward and reverse mutation rates, sensitivity to DNA damage, transcriptional activity, growth defects, protein mislocalization, and intra- and inter-chromosomal recombination.

Because all MMR genes have orthologues in *S. cerevisiae*, human sequence variants can frequently be introduced into the corresponding yeast gene, or the endogenous yeast gene can be replaced with its human orthologue carrying the variant of interest [170].

In the context of *MLH1* and *MSH2* genes, several researchers used forward and reverse mutation assays for more than 25 years. While forward mutation assays can detect loss of gene function by selecting for resistance or loss of growth, reverse mutation assays detect restored function by selecting for growth on media lacking a required nutrient or containing a toxic analogue [169,171,172,173,174,175,176,177].

#### 6.2.3. Yeast—Dominant Mutator Effect/Phenotype

Expression of wild-type human *MLH1* (*hMLH1*) in *S. cerevisiae* can lead to a dominant mutator phenotype when expressed at high levels. This effect is likely due to competition between hMLH1 and endogenous yeast MLH1 protein partners, thereby disrupting the yeast MMR pathway. Consequently, if an *hMLH1* variant results in a functionally compromised protein, it is less likely to interfere with the yeast MLH1 and the MMR pathway, allowing the yeast to maintain its natural repair activity.

In 1998, Shimodaira et al. developed a functional assay system to investigate 27 *hMLH1* variants using both forward and reverse mutation approaches. This system exploits the dominant mutator effect (DME) caused by high-level expression of *hMLH1* in yeast. The authors expressed *hMLH1* cDNA in a series of MMR-deficient and MMR-proficient *S. cerevisiae* strains using plasmids carrying the *hMLH1* gene. These included a low-copy centromeric vector (pCI-ML10) and a high-copy vector (pCLML9), allowing assessment of variant function based on the degree of MMR disruption.

The strains expressing the variants were then evaluated to determine mutation rates using two assays: reversion of the *hom3-10* allele (reverse mutation), or resistance to canavanine (Can^r^) (forward mutation). The *HOM3* gene in this allele (*hom3-10*) is inactivated by the insertion of an extra thymine (T) in a stretch of six T residues. A single T deletion restores the correct reading frame, allowing the strains to grow in the absence of threonine. Regarding Can^r^, *CAN1* encodes an arginine permease that is essential for the uptake of canavanine into the cell. When this gene is inactivated by mutation, cells become resistant to canavanine and are able to grow. Both assays exhibit increased reversion rates when MMR is defective [171].

Another DME study by Takahashi et al. evaluated 101 *MLH1* variants (99 missense, 1 nonsense (p.Trp714*) and a 3 bp deletion (p.Lys618del). The authors employed three different reporter-based methods—using GFP, ADE2, and LacZ—within the framework of the DME (reverse mutation) approach and compared data with a human cell-based MMR assay. They used human colon cancer HCT116 cells, which are deficient in *MLH1* and *PMS2*, which were transiently transfected to coexpress *PMS2* with either wild-type *MLH1* or the test variant under study. Among the variants analyzed, 26 missense variants, as well as p.Trp714* and p.Lys618del, tested negative in all three DME assays and exhibited loss of MMR activity in the MMR assay (with MMR activity below 60%). In contrast, 36 variants were positive across all three assays and showed MMR activity above 60%, indicating no loss of MMR function according to the thresholds defined by the author. The remaining variants did not show full concordance across the four assays [169]. Table 1 lists a selection of these functionally tested MLH1 variants and compares the test result with that from other studies, and indicates their classification that we obtained according to the most recent MMR classification guidelines.

Regarding *MSH2* variants, DME assays have also been applied by Drotschmann et al. who analyzed 7 variants using *LYS2* and Can^r^ assays in *S. cerevisiae*. This was followed by further studies such as the work of Gammie et al., who examined 54 *hMSH2* variants—using *URA3*, Can^r^ and two-hybrid assays that revealed impaired biological function in at least 21 of the tested variants [172,175].

#### 6.2.4. Yeast Two-Hybrid Assay

Developed by Fields and Song in 1989, the two-hybrid system allows the study of interactions between two hybrid proteins expressed in *S. cerevisiae* [182]. The assays are based on two hybrid proteins, one consisting of the GAL4 DNA-binding domain fused to a protein of interest (X), and another comprising the GAL4 activation domain fused to a second protein (Y). When X and Y interact, the proximity of the GAL4 domains restores transcriptional activity at a reporter gene containing the GAL4 recognition motif UAS_G_. Later improvements include the use of *LexA* and *VP16* fusion protein systems.

Several studies applied two-hybrid assays in the context of MMR genes. In order to identify the essential regions mediating protein–protein interactions, cDNA constructs with 16 different deletions in *hMLH1* or 27 in *hPMS2* were fused to either the LexA DNA-binding domain or the VP16 transcriptional activation domain, and revealed that the minimal regions required for interaction were residues 492–742 in MLH1 and residues 612–674 in PMS2 [172,183]. Subsequently, the same authors investigated the effects of 47 *hMLH1* variants (including 32 missense, 11 frameshift, 3 nonsense, and 1 in-frame variant) on the interaction with both PMS2 and EXO1. This study provided in vivo evidence for how specific missense variants in *MLH1* can disrupt key protein–protein interactions critical for the proper function of the MMR pathway [178]. A second set of studies used a two-hybrid approach to study the MSH2 protein, including missense variants or its interaction with MSH6 [172,183,184].

#### 6.2.5. In Vivo Assays in Mammalian Cells Based on 6-Thioguanine (6-TG) Resistance

The DNA-damaging agent 6-TG is a cell-permeable purine analog incorporated into DNA during replication in place of dGTP. After incorporation into DNA, 6-TG can be methylated into 6-methylthioguanine (6-meTG) by cellular methyltransferases using S-adenosylmethionine as the methyl donor. In the next replication cycle, 6-meTG can pair with either thymine or cytosine, and the resulting mismatch will be recognized by the MMR machinery [185]. However, the MMR system is unable to repair these lesions, leading to replication arrest and, ultimately, induction of cell death [164,186], the reason why 6-TG can be used in the clinic as a chemotherapeutic drug.

Accordingly, if the missense variant under study retains biological MMR function, the cells will be sensitive to 6-TG, but if it impairs MMR, the cells will tolerate and survive the presence of 6-TG. This selection strategy has been widely employed in studies using mESCs to study *MLH1*, *MSH6*, *MSH2* and *PMS2* variants [163,179,187,188,189].

6-TG-based MMR assays were also employed following oligonucleotide-directed mutation screening (ODMS) in mESC cells. ODMS is an oligonucleotide-targeting s-dm approach that uses locked nucleic acid (LNA)-modified oligos to introduce up to three base pair substitutions in mESCs, at endogenous genomic loci [190,191,192]. The introduced variant MMR genes are thus expressed at physiological levels. With this approach, Houlleberghs et al. generated several *Mlh1* hemizygous mESC lines that were subsequently exposed to 6-TG to determine whether the variant abrogated MMR. In these cells, a puromycin resistance gene (+PUR) was inserted upstream of the *Mlh1* allele (*Mlh1*^+PUR/ΔmESC^) to prevent the survival of MMR-deficient cells arising from spontaneous loss of the wild-type *Mlh1*. Subsequently, cells resistant to both 6-TG and puromycin were sequenced to confirm the presence of the introduced variants. This three-step s-dm approach, achieved a sensitivity greater than 95% and a specificity equal to or higher than 91%, and identified 31 pathogenic variants among 51 VUS [179].

#### 6.2.6. Methylation Tolerance (MT)-Based Functional Assays

The MT functional assay is based on exposure of cells to the methylating agent N-Methyl-N′-Nitro-N-Nitrosoguanidine (MNNG) followed by analysis of MMR-dependent cell death (apoptosis). Bouvet et al. developed and applied this method to characterize *MLH1* and *MSH2* variants (88 in total, with 40 used as controls). These variants were introduced by s-dm into the expression vectors pIRES or pVAX1, which contained the wild-type cDNA of MLH1 and MSH2, respectively. The pIRES vector also carried mCherry as a reporter gene, while the pVAX1 vector included GFP as a reporter. The resulting bicistronic plasmids were transfected into the human CRC cell lines HCT116 (MLH1-deficient), HCT116-ch3 (MLH1-proficient), or LoVo (MSH2-deficient). After sorting of fluorescent cells, the vector-positive cells were incubated with MNNG for 12 to 14 days. Subsequently, the cells were fixed, stained, and colonies were counted to calculate the survival fraction, as assessed by clonogenic growth assays. Among 48 VUS studied, cells expressing one of 12 pathogenic *MLH1* or *MSH2* variants survived at higher rates, similar to MLH1-deficient HCT116 and MSH2-deficient LoVo cells. In contrast, expression of one of 28 benign variants that did not compromise the MMR pathway led to decreased survival following MNNG exposure. However, 8 variants yielded inconclusive results and therefore remained classified as VUS [180].

This assay type was more recently combined with a more complex strategy for performing s-dm via Clustered Regularly Interspaced Short Palindromic Repeats—(CRISPR)-Cas9 gene editing, (reviewed in [193]). Briefly, single guide RNAs (sgRNAs) bound to the CRISPR protein direct the DNA endonuclease Cas9 to the desired 18 to 20 nucleotide long genomic target location, where Cas9 introduces double-strand breaks (DSBs). After cleavage, the resulting DSBs are repaired by cellular mechanisms such as non-homologous end joining (NHEJ) or homology-directed repair (HDR) [193]. Single-stranded deoxyoligonucleotides (ssODNs) can be delivered to provide the templates containing the variants to be introduced by recombination [194]. Meanwhile, various systems for gene editing are available, such as plasmid-based CRISPR-Cas9 expression, ribonucleoprotein (RNP) complexes consisting of Cas9 protein and sgRNA, and Cas9 mRNA delivered into cells together with sgRNA [195].

Gene editing has been successful in different cell lines and stem cells and have generated variants at the endogenous chromosomal locus with its natural regulatory mechanisms. This also allows to assess the effect of variants at the RNA level, including potential splicing or mRNA stability issues [181].

Regarding functional studies of MMR genes, Rath et al. constructed 20 different *MSH2* clones/cell lines using hESCs (H1) as the cell model. In these clones, 9 *MSH2* VUS, 1 variant associated with cancer in mice, and 10 control variants (5 already classified as benign and the other 5 as pathogenic) were introduced at the endogenous *MSH2* locus in a homozygous state, thereby leading to the simultaneous knockout of the wild-type *MSH2* gene [194].

To that end, the authors used plasmid vectors encoding the sgRNA and Cas9 to introduce the genomic DSBs near the sites of *MSH2* variants, together with ssODNs containing the specific test variants. After confirming that the clones carried the variants at the expected location, the biological impact of these variants was evaluated. For this, they analyzed splicing assay (to evaluate possible impact on exon inclusion), immunoblotting (to analyze the steady-state protein levels), MSI analysis (using the MSI loci BAT-26 and NR-27), NGS (to assess the mutation rate in 501 genes), and MT assays with MNNG treatment. Clones with pathogenic variants and the *MSH2* knockout exhibited a survival advantage when exposed to MNNG, compared to hESCs with wild-type *MSH2* and to clones with benign variants. Among the analyzed VUS, four variants (p.Asp603Val, p.Gly674Ala, p.Ser723Phe, and p.Asp748Tyr) were considered pathogenic, two (p.His639Arg and p.Ser516Ile) remained classified as VUS due to the observation of an intermediate phenotype (MSI-L and MT assays), and the remaining variants were classified as benign [181].

A follow-up study using recombinant Cas9 or Cas12a enzymes pre-incubated with variant-specific guide RNA, assessed 21 *MLH1* VUS, along with 22 isogenic control cell lines (11 benign and 11 pathogenic), and an *MLH1* knockout cell line. The authors also performed multiple assays, including an exon inclusion assay, immunoblotting (to evaluate steady-state protein levels of MLH1 and mPMS2), MT assays with MNNG and MSI analysis using loci BAT-26 and NR-27, as well as NR-21, NR-22, and BAT-25 [181,194].

Following the above-described assay panel, only five variants (p.Cys39Arg, p.Asp36Asn, p.Leu73Pro, p.Ala111Pro and p.Gly244Val) were correctly classified as likely pathogenic according to the InSiGHT classification system. Among the remaining variants, nine remained classified as VUS, eight as likely benign or benign. Although some of the likely pathogenic (p.Leu73Pro, p.Ala111Pro and p.Gly244Val) or likely benign (p.Asn338Ser, p.Gly454Arg and p.Arg474Gln) variants were consistent with previous studies [159,169,179,181], this was not the case for other variants, such as p.Arg9Trp, p.Gln542Leu and p.Asn64Ser, indicating that functional studies need to be accompanied by InSiGHT classification.

Supplementary Table S3 summarizes and compares the described functional assay systems regarding their aim, biological system, read-out, advantages, and limitations. InSiGHT ClinGen and CanVIG-UK classification guidelines attribute high scores to results obtained in cell-free in vitro mismatch repair activity and human cell-based assays.

### 6.3. High-Throughput Studies Using Multiplexed Assays of Variant Effect (MAVE)

MAVE is a high-throughput experimental strategy that integrates diverse methodologies, including saturation s-dm (variant library preparation), delivery of the library or set of variants into a cellular model or assay system, followed by variant scoring (phenotypic readout and selection-based approaches of variants, sequencing and demultiplexing techniques, and computational analysis pipelines for functional interpretation), and were comprehensively reviewed elsewhere [196,197,198,199,200,201].

Kitzman et al. coined the term MAVE in 2017 for assays with a comprehensive and systematic approach to investigate the functional impact of genetic variations in proteins or nucleic acids [200]. Previously called as Deep Mutational Scanning (DMS), MAVE was first employed more than a decade ago, in 2010, to study the PDZ domain [202] or WW domain of YAP1 [203].

In the context of LS, Ollodart et al. employed *S. cerevisiae* strains to evaluate a library comprising 185 missense variants, of which 28 were of known classification and 157 had been classified as VUS. They used mutation rate and Can^r^ as functional readouts and concluded that 50 variants were pathogenic, based on differences in mutation rates observed between strains expressing the variants and those expressing wild-type protein [204]. The assay also confirmed the classification of the 28 known variants. In parallel, Jia et al. assessed the remarkable number of 17,746 missense variants, representing approximately 94% of all possible missense variants in *MSH2*, using human cell lines (HAP1, HEK293, and 293T/17) as models and a 6-TG resistance assay as a selection-based method. They reported that approximately 10–11% of the variants were pathogenic [81,196,204,205].

More recently, Herger et al. performed a MAVE analysis of *MLH1* variants and utilized prime editing—an approach using Cas9 nickases fused to reverse transcriptase domains enabling the introduction of any short variant into the genome [201,206]. They performed variant editing in the near haploid and MMR-proficient HAP1 cell line, using a dominant-negative MLH1 variant as control, followed by 6-TG selection to identify impaired MMR function. The authors introduced 2696 prime-editing guide RNAs encoding 598 variants, including 22 nonsense multi-nucleotide variants (MNVs) and 96% of all possible single-nucleotide variants (SNVs) within a 200 bp region of MLH1 exon 10 and its flanking intronic regions. They found a cluster of LoF missense variants near the end of exon 10, a highly conserved β-sheet in the DNA mismatch repair protein C-terminal domain of MLH1 (see yellow domain in Figure 2). Additionally, they selected from ClinVar 874 non-coding variants smaller than 10 nucleotides (771 SNVs, 69 deletions, 25 insertions, and 9 MNVs) distributed across 60 kb of MLH1. Although most non-coding variants that scored as LoF in HAP1 cells were also predicted by the SpliceAI algorithm, the assays also identified LoF variants deeper in introns, in the 5′-UTR, and upstream of the transcriptional start site, i.e., in genomic regions that lack definitive interpretations in ClinVar [201]. It should be noted that a current limitation of these MAVE-derived data is their validation in cell types that do not fully represent the in vivo context of colon cancer.

## 7. Conclusions and Future Perspectives

In parallel with the increasing numbers of CRC cases, it may be expected that the number of LS diagnoses will also rise in the next decade [207]. The current shift in diagnosis towards multi-omics approaches integrating genomic, transcriptomic, proteomic, and metabolomic levels, will become standard for tailoring the appropriate and more personalized therapeutic strategy for each patient, both in sporadic CRC and LS. This calls for a more systematic determination of the MMR gene variants present in non-caucasian populations, because most of the currently available genetic data on MMR derive from European and decedent populations.

In the light of rapidly evolving molecular tools and functional assays, this review also highlights how we can expect an improved interpretation of mismatch repair variants for the corresponding clinical decision-making. First, the growing application of artificial intelligence (AI) in in silico analysis tools, such as SpliceAI, is expected not only to improve genetic variant classification, but also cancer diagnosis and therapeutic outcome prediction [28]. Second, improved identification of LS cases and respective therapeutic options can be expected to result from emerging molecular tools that associate miRNA expression analysis, as well as epigenetic changes affecting DNA methylation or histone deacetylation [10]. Furthermore, the aforementioned proposal to redefine LS based on the affected gene, may lead to the inclusion of certain LLS cases attributable to pathogenic germline variants in other genes that disrupt proper MMR function, such as the previously mentioned *MCM 9* [45,46,65,208]. Third, additional candidate genes or genomic regions associated with LS can be expected to emerge, including those identified through genome-wide association studies (GWAS), expression quantitative trait loci (eQTL) analyses, or methylome-wide association studies (MWAS) [28,209,210]. The SNPs identified in these studies likely represent low-penetrance genetic modifiers that individually confer only modest increases in risk, but may act synergistically—when combined in polygenic risk scores—to elevate overall LS penetrance [211,212].

Finally, it is also foreseeable that emerging approaches and technologies, such as whole genome and long-read sequencing, will overcome the limitations of currently employed screening methods, in particular of technically challenging variants in MMR genes, that might include inversions (e.g., exons 1–7 and 2–6 in *MSH2*), structural variants (e.g., *MLH1–LRRFIP2* rearrangements), deep intronic MMR variants or mosaicism cases, that may include cases previously classified as LLS [45].

In light of these developments, the (re)classification of MMR gene variants and existing clinical guidelines for LS (e.g., colonoscopy surveillance interval), will continue to require international harmonization (as it recently happened with MMR variant classification guidelines), to ensure precise diagnoses and appropriate clinical management. This continuous improvement of genomic technologies and screening methods as well as the increased data generation represents a major challenge for clinicians and laboratories that need to remain constantly updated.

Before the era of MAVEs, the majority of the VUS that have been assessed by functional studies were selected in a “reactive” approach after they had been identified in patients [213]. However, the recent application of high-throughput MAVE capable of targeting all possible missense and non-coding variants of *MSH2* and *BRCA1* [214] have changed this approach. This new technology brings prospect that in the (near) future similar strategies may be applied to all MMR genes (in case of *PMS2*, the number of pseudogenes, particularly *PMS2CL*, will hamper such large-scale variant screening). This calls for guidelines establishing the minimum required information, along with recommendations on how MAVE studies should be reported, collected and interpreted for clinical use [215,216], in particular their value for variant classification. Simultaneously, an international alliance has also been established aiming to coordinate efforts of creating an atlas encompassing all single nucleotide variants (initially) based on functional assessment, complementing results from in silico tools such as AlphaMissense and Alphafold, which covers all missense variants across all known human genes and proteins [217,218,219]. This will be essential for advancing diagnosis but may also lead to a better understanding of how gene pathways work and proteins act in different cellular contexts.

### Limitations of the Presented Review

The narrative nature of this review implies the possibility of a bias in the selected literature on which it was based. Furthermore, the presented conclusions are conditioned by rapid changes in guidelines for variant classification and databases, by a lack of harmonization among functional assays that analyze VUS, and by the limited clinical readiness and validation of some high-throughput approaches.

## Figures and Tables

**Table 1:** Simplified overview of the functional analyses and classification of selected *hMLH1* missense variants found in LS patients. The MLH1 protein domain colours correspond to those in Figure 2. The 31 missense variants functionally analyzed by Takahashi et al. (2007) [169] are compared across 12 additional functional studies, alongside the variant classifications according to InSiGHT ClinGen and CanVIG-UK (blue-boxed criteria). Square colours indicate MMR activity: Pink-Deleterious, Green-proficient, Grey-uncertain, White-not tested. Letters indicate variant classification: B-Benign, P-Pathogenic, LP-Likely Pathogenic, LB-Likely Benign, VUS-Variant of uncertain significance. Numbers in square brackets correspond to the reference list. T117M and I219V (in bold) were used as pathogenic/deficient and benign/proficient MMR activity controls, respectively. The completely annotated dataset including numerical values of in silico classifications, publication and algorithm references is available as Supplementary Tables S1 and S2.

Functional Studies															ClinGen InSiGHT and CanVIG-UK Classification		
Missense Variants Analyzed by Takahashi et al, 2007 [169]			Yeast-Based Assays					In Vitro MMR Assays				hCRC/ mESC Assays					
Domain (aa)	Missense	MMR Activity (%)	[171]	[176]	[174]	[178]	[177]	[159]	[158]	[167]	[168]	[179]	[180]	[181]	Spl AI	HCI ppp	Class
ATPase (26–139)	P28L (c.83C>T)	9.2															P
	N38D (c.112A>G)	0															LP
	G54E (c.161G>A)	47.9															VUS
	N64S (c.191A>G)	36.6															VUS
	C77Y (c.230G>A)	11.2															P
	F80V (c.238T>G)	23.7															VUS
	T82I (c.245C>T)	27.2															P
	K84E (c.250A>G)	22.5															P
	R100P (c.299G>C)	0															LP
	**T117M (c.350C>T)**	34.8															P
CTD (216–335)	R217C (c.649C>T)	64.8															B
	**I219V (c.655A>G)**	60.7															B
	R226L (c.677G>T)	39.2															P
	R265C (c.793C>T)	55															P
	E268G (c.803A>G)	78.9															VUS
	K286Q (c.856A>C)	78.6															VUS
	S295G (c.883A>G)	75.5															P
	D304V (c.911A>T)	0															LP
	H329P (c.986A>C)	25.7															P
-	A492T (c.1474G>A)	65.3															VUS
MMR MLH1-CTD (502–756)	V506A (c.1517T>C)	67.6															VUS
	N551T (c.1652A>C)	78.9															VUS
	E578G (c.1733A>G)	51.2															LB
	L588P (c.1763T>C)	68.3															VUS
	L622H (c.1865T>A)	69.2															P
	R659Q (c.1976G>A)	79.7															LP
	T662P (c.1984A>C)	64															LP
	E663D (c.1989G>T)	68.5															P
	A681T (c.2041G>A)	69.8															P
	V716M (c.2146G>A)	75.1															B
	H718Y (c.2152C>T)	84.5															B

## Data Availability

The original contributions presented in this study are included in the article and Supplementary Material. Further inquiries can be directed to the corresponding authors.

## Supplementary Materials

The following supporting information can be downloaded at https://www.mdpi.com/article/10.3390/biomedicines14061312/s1, Legend S1: Detailed legend to Figure 2; Table S1: Detailed description of 31 MLH1 VUS regarding functional assays, in silico prediction and variant classification; Table S2: InSiGHT ClinGen and CanVIG-UK scores attributed to the 31 MLH1 VUS presented in Table 1. Table S3: Comparison of functional assay systems regarding their aim, biological system, read-out, advantages, and limitations.

## Abbreviations

The following abbreviations are used in this manuscript:

CIMRA	Cell-free in vitro MMR activity
CNV	Copy number variant
CRC	Colorectal cancer
EC	Endometrial cancer
LLS	Lynch-like syndrome
LoF	Loss of function
LS	Lynch syndrome
MMR	Mismatch repair
MSI	Microsatellite instability
VUS	Variant of uncertain significance
s-dm	Site-directed mutagenesis
MAVE	Multiplexed assays of variant effect
