# Supplementary material for: Lynch Syndrome: An Update of Underlying Molecular Mechanisms, Phenotypes and Methods to Classify Variants of Uncertain Significance
Source: Biomedicines. 2026 Jun 9;14(6):1312. doi: 10.3390/biomedicines14061312 (PMC13297208; doi:10.3390/biomedicines14061312)
Supplement: Supplementary file 1 [file biomedicines-14-01312-s001.zip › biomedicines-4309777-supplementary.pdf]

Review “Lynch Syndrome: an update of underlying molecular mechanisms, phenotypes and methods to classify variants of uncertain significance”

Supplementary Legend S1

Detailed legend to Figure 2. Protein structure models of heterodimers formed by human MSH2 and MLH1.

The predicted 3D models were generated with ChimeraX daily software based on PDB files obtained from the Alphafold database. The amino acid ranges for each protein domain were determined based on information from Dechipher and Pfam.

The figure allows visualizing the following structural similarities among the indicated paralogous MMR proteins: (**A**) the MutS complex partners MSH2, MSH3, and MSH6 share the DNA mismatch-binding domain (MutS domain I; orange), the connector domain (MutS domain II; dark yellow), the lever domain (MutS domain III; purple) and the ATPase domain (MutS domain V; green). The clamp domain (MutS domain IV; light pink) is only present in MSH2 and MSH6, while the Pro-Trp-Trp-Pro motif that binds histone H4 methylated at lysine 20 (PWWP domain; dark cyan) is present only in MSH6.

Concerning the MutL complex partners (**B**), MLH1, PMS2, MLH3, and PMS1 share the histidine kinase-like ATPase domain/HATPase C 3/Hsp90-like ATPase (hot pink) and the DNA mismatch repair protein C-terminal domain (yellow). The MLH1 C-terminal domain (cornflower blue) is present in MLH1, PMS2, and MLH3, but not in PMS1. PMS1 is the only MMR protein that contains a high-mobility group (HMG)-box domain (light cyan), which is involved in DNA binding.

Supplementary Table S1. Detailed description of 31 MLH1 VUS regarding functional assays, *in silico* prediction and variant classification

Functional studies																					
Missense variants analyzed by Takahashi et al, 2007 [169]				Yeast-based assays					*in vitro* MMR assays				hCRC/ mESC assays			*In silico* prediction				Variant Classification	
**Domain (aa)**	**Missense**	**DME (3 assays)**	**MMR activity (%)**	**Shimodaira et al., 1998 [171]**	**Wanat et al, 2006 [176]**	**Vogelsang et al., 2009 [174]**	**Kondo et al, 2003 [180]**	**Hardt et al, 2011 [177]**	**Drost et al., 2010 [159]**	**Drost et al., 2019 [158]**	**Raevaara et al 2005 [167]**	**González-Acosta et al., 2020 [168]**	**Houllebergh et al., 2020 [184]**	**Bouvet et al., 2019 [191]**	**Rath et al., 2022 [195]**	**REVEL**	**Alpha Missense**	**SpliceAI**	**HCI ppp**	**InSiGHT ClinGen and CanVIG-UK**	**ClinVar (nº of stars)**
**ATPase (26-139)**	P28L (c.83C>T)	1+	9,2	Not tested	Deficient	Not tested	Deficient	Deficient	Not tested	Not tested	Deficient	Not tested	Not tested	Not tested	Not tested	0.85 Del.	0.983 Del.	0,02 DL Benign	0.9265 Del. Mod.	P	P (3*)
	N38D (c.112A>G)	2+	0	Not tested	Not tested	Not tested	Not tested	Not tested	Not tested	Not tested	Not tested	Not tested	Deficient	Not tested	Not tested	0.93 Del.	0.999 Del.	0.1 DL Benign	0.9385 Del. Mod.	LP	Not reported
	G54E (c.161G>A)	1+	47.9	Not tested	Not tested	Not tested	Not tested	Not tested	Not tested	Not tested	Not tested	Not tested	Not tested	Not tested	Not tested	0.92 Del.	0.995 Del.	0.01 AG Benign	0.9591 Del. Mod.	VUS (hot)	Not reported
	N64S (c.191A>G)	1+	36.6	Not tested	Deficient	Not tested	Not tested	Uncertain	Not tested	Deficient	Not tested	Not tested	Not tested	Not tested	Proficient	0.93 Del.	0.17 Benign	0 Benign	0.7179 Del. Spp.	VUS (hot)	VUS (1*)
	C77Y (c.230G>A)	2+	11.2	Deficient	Deficient	Not tested	Deficient	Not tested	Not tested	Not tested	Not tested	Not tested	Not tested	Not tested	Not tested	0.99 Del.	0.998 Del.	0.01 AL Benign	0.8804 Del. Mod.	P	P (3*)
	F80V (c.238T>G)	1+	23.7	Not tested	Not tested	Not tested	Not tested	Deficient	Not tested	Not tested	Deficient	Not tested	Not tested	Not tested	Not tested	0.97 Del.	0.998 Del.	0.01 AL Benign	0.8274 Del. Mod.	VUS (hot)	LP (3*)
	T82I (c.245C>T)	2+	27.2	Not tested	Not tested	Not tested	Not tested	Not tested	Not tested	Not tested	Not tested	Not tested	Not tested	Not tested	Not tested	0.87 Del.	0.997 Del.	0.04 AL Benign	0.9539 Del. Mod.	P	P (3*)
	K84E (c.250A>G)	2+	22.5	Not tested	Not tested	Not tested	Not tested	Deficient	Not tested	Not tested	Deficient	Not tested	Deficient	Not tested	Not tested	0.95 Del.	0.999 Del.	0 Benign	0.9043 Del. Mod.	P	LP (3*)
	R100P (c.299G>C)	2+	0	Not tested	Deficient	Not tested	Not tested	Not tested	Not tested	Not tested	Not tested	Not tested	Not tested	Not tested	Deficient	0.89 Del.	0.999 Del.	0.06 DL Benign	0.9699 Del.Mod.	LP	P (3*)
	**T117M (c.350C>T)**	0+	34.8	Deficient	Not tested	Deficient	Deficient	Not tested	Deficient	Not tested	Not tested	Not tested	Not tested	Deficient	Deficient	0.87 Del.	0.883 Del.	0 Benign	0.893 Del. Mod.	**P**	P (3*)
**CTD (216-335)**	R217C (c.649C>T)	2+	64.8	Not tested	Not tested	Not tested	Proficient	Not tested	Not tested	Not tested	Not tested	Not tested	Not tested	Not tested	Not tested	0.84 Del.	0.382 Uncertain	0.01 DL Benign	0.5783 Uncertain	B	Conflicting/LB(1*)
	**I219V (c.655A>G)**	**3+**	**60.7**	Proficient	Not tested	Proficient	Proficient	Not tested	Proficient	Not tested	Proficient	Proficient	Not tested	Proficient	Proficient	0.29 Benign	0.059 Benign	0 Benign	0.0011 Benign	**B**	B (3*)
	R226L (c.677G>T)	1+	39.2	Not tested	Not tested	Not tested	Not tested	Not tested	Not tested	Not tested	Not tested	Not tested	Not tested	Not tested	Not tested	0.9 Del.	0.818 Del.	0.45 DL SA mod.	0.8626 Del. Mod.	P	LP (3*)
	R265C (c.793C>T)	2+	55	Not tested	Not tested	Not tested	Not tested	Not tested	Deficient	Not tested	Not tested	Not tested	Not tested	Not tested	Not tested	0.97 Del.	0.986 Del.	0.15 AL Uncertain	0.9373 Del. Mod.	P	P (3*)
	E268G (c.803A>G)	2+	78.9	Not tested	Not tested	Not tested	Not tested	Not tested	Not tested	Uncertain	Not tested	Not tested	Not tested	Not tested	Proficient	0.93 Del.	0.623 Uncertain	0 Benign	0.7087 Del. Spp.	VUS (cold)	B (3*)
	K286Q (c.856A>C)	2+	78.6	Not tested	Not tested	Not tested	Not tested	Not tested	Not tested	Not tested	Not tested	Not tested	Not tested	Not tested	Uncertain	0.91 Del.	0.801 Del.	0.04 DL Benign	0.7401 Del. Spp.	VUS (cold)	VUS (2*)
	S295G (c.883A>G)	2+	75.5	Not tested	Not tested	Not tested	Not tested	Not tested	Not tested	Not tested	Not tested	Not tested	Not tested	Not tested	Not tested	0.89 Del.	0.258 Benign	0.27 DL SA mod.	0.4807 Uncertain	P	P (3*)
	D304V (c.911A>T)	1+	0	Not tested	Not tested	Not tested	Not tested	Not tested	Not tested	Deficient	Not tested	Not tested	Deficient	Not tested	Not tested	0.95 Del.	0.994 Del.	0 Benign	0.8911 Del. Mod.	LP	LP (3*)
	H329P (c.986A>C)	1+	25.7	Not tested	Not tested	Not tested	Not tested	Not tested	Not tested	Not tested	Proficient	Not tested	Not tested	Not tested	Not tested	0.82 Del.	0.87 Del.	0 Benign	0.1457 Uncertain	P	P (3*)
-	A492T (c.1474G>A)	2+	65.3	Deficient	Not tested	Not tested	Proficient	Not tested	Not tested	Not tested	Not tested	Not tested	Not tested	Not tested	Not tested	0.5 Uncertain	0.143 Benign	0 Benign	0.0244 Benign	VUS (cold)	VUS (1*)
**MMR MLH1-CTD (502-756)**	V506A (c.1517T>C)	2+	67.6	Deficient	Not tested	Not tested	Proficient	Not tested	Not tested	Not tested	Not tested	Not tested	Not tested	Not tested	Proficient	0.76 Del.	0.865 Del.	0 Benign	0.3487 Uncertain	VUS (cold)	LP(1*)
	N551T (c.1652A>C)	0+	78.9	Not tested	Not tested	Not tested	Not tested	Not tested	Not tested	Not tested	Not tested	Not tested	Deficient	Not tested	Not tested	0.8 Del.	0.538 Uncertain	0.02 DL Benign	0.1005 Benign	VUS	VUS (1*)
	E578G (c.1733A>G)	2+	51.2	Deficient	Not tested	Not tested	Proficient	Not tested	Proficient	Proficient	Not tested	Not tested	Not tested	Not tested	Proficient	0.8 Del.	0.182 Benign	0.09 AG Benign	0.0129 Benign	LB	B (3*)
	L588P (c.1763T>C)	1+	68.3	Not tested	Not tested	Not tested	Not tested	Not tested	Not tested	Not tested	Not tested	Not tested	Deficient	Not tested	Not tested	0.95 Del.	0.961 Del.	0 Benign	0.9472 Del. Mod.	VUS	VUS (1*)
	L622H (c.1865T>A)	0+	69.2	Not tested	Deficient	Not tested	Not tested	Not tested	Not tested	Deficient	Not tested	Deficient	Not tested	Deficient	Not tested	0.97 Del.	0.975 Del.	0 Benign	0.8294 Del. Mod.	P	P (3*)
	R659Q (c.1976G>A)	2+	79.7	Not tested	Not tested	Deficient	Not tested	Not tested	Not tested	Not tested	Proficient	Not tested	Not tested	Not tested	Not tested	0.8 Del.	0.412 Uncertain	0 Benign	0.7507 Del. Spp.	LP	VUS (1*)
	T662P (c.1984A>C)	0+	64	Not tested	Not tested	Not tested	Not tested	Not tested	Not tested	Not tested	Not tested	Not tested	Not tested	Not tested	Not tested	0.91 Del.	0.863 Del.	0.02 DG Benign	0.972 Del. Mod.	LP	LP (3*)
	E663D (c.1989G>T)	2+	68.5	Not tested	Not tested	Not tested	Not tested	Not tested	Not tested	Not tested	Not tested	Not tested	Not tested	Not tested	Not tested	0.67 Del.	0.817 Del.	0.92 DG SA str	0.3442 Uncertain	P	P (3*)
	A681T (c.2041G>A)	0+	69.8	Deficient	Proficient	Not tested	Deficient	Not tested	Not tested	Proficient	Proficient	Uncertain	Not tested	Deficient	Not tested	0.83 Del.	0.735 Uncertain	0 Benign	0.0273 Benign	P	P (3*)
	V716M (c.2146G>A)	1+	75.1	Not tested	Not tested	Not tested	Not tested	Not tested	Proficient	Not tested	Proficient	Proficient	Not tested	Proficient	Proficient	0.52 Uncertain	0.209 Benign	0 Benign	0.3241 Uncertain	B	B (3*)
	H718Y (c.2152C>T)	2+	84,5	Not tested	Proficient	Not tested	Proficient	Not tested	Not tested	Not tested	Not tested	Not tested	Not tested	Proficient	Proficient	0.9 Del.	0.225 Benign	0.01 AG Benign	0.1729 Uncertain	B	B (3*)

Supplementary Table S1. Detailed description of 31 *MLH1* VUS regarding functional assays, in silico prediction and variant classification. Takahasi et al (2007) functional assays were performed both, in yeasts strains, using three different reporter-based methods (GFP, ADE2, and LacZ) based on dominant mutator effect (DME), and in MLH1-deficient human cells (HCT116, considering restoration of MMR proficiency if MMR activity > 60%). The MLH1 protein domain colors correspond to those in Fig. 2. CTD -C terminal domain, Del-Deleterious, mod-moderate, str-strong, Spp- supporting, SA -Splicing-altering, P-Pathogenic, LP- Likely Pathogenic, VUS-Variant of uncertain significance, LB-Likely Benign and B-Benign, AG -Acceptor gain, DL -donor loss, SA - splice altering. T117M and I219V were used as pathogenic/deficient and benign/proficient MMR activity controls, respectively. Functional studies and in silico prediction tools labeled in blue are recommended to use by InSiGHT ClinGen and CanVIG-UK MMR specific guidelines, whenever applicable. The in silico tools range scores, where applied according to each in silico tool and the ClinGen InSiGHT/CanVIG-UK guidelines intructions. "Cold" VUS and "Hot" VUS refer to variants classified with scores between 0 and 2 points, or 4 to 5 points, respectively.

Supplementary Table S2. InSiGHT ClinGen and CanVIG-UK scores attributed to the 31 MLH1 VUS presented in Table 1

		Takahashi et al, 2007 [169]		Reported ClinVar	InSiGHT ClinGen and CanVIG-UK, v1.0.0 applied criteria																		Points	InSiGHT ClinGen and CanVIG-UK Classification v1.0.0
Domain (aa	Missense	DME (3 assays)	MMR activity (%)	ClinVar	PVS1	PS1	PS2	PS3	PM2	PM3	PM5	PP1	PP3	PP4	BA1	BS1	BS2	BS3	BS4	BP4	BP5	BP7	Total	
ATPase (26-139)	P28L (c.83C>T)	1+	9.2	P (3*)	0	0	0	2	1	0	1	0	2	4	0	0	0	0	0	0	0	0	10	Pathogenic (1 str criteria + 2 mod +2 spp)
	N38D (c.112A>G)	2+	0	Not reported	0	0	0	2	1	0	2	0	2	0	0	0	0	0	0	0	0	0	7	Likely Pathogenic (3 mod criteria + 1 spp)
	G54E (c.161G>A)	1+	47.9	Not reported	0	0	0	2	1	0	0	0	2	0	0	0	0	0	0	0	0	0	5	VUS (hot) (2 mod criteria + 1 spp)
	N64S (c.191A>G)	1+	36.6	VUS (1*)	0	0	0	1	0	0	0	1	1	2	0	0	0	0	0	0	0	0	5	VUS (hot) (1 mod criteria + 3 spp)
	C77Y (c.230G>A)	2+	11.2	P (3*)	0	0	0	1	1	0	2	0	2	4	0	0	0	0	0	0	0	0	10	Pathogenic (1 str criteria + 2 mod +2 spp)
	F80V (c.238T>G)	1+	23.7	LP (3*)	0	0	0	1	1	0	1	0	2	0	0	0	0	0	0	0	0	0	5	VUS (hot) (1 mod criteria + 3 spp criteria)
	T82I (c.245C>T)	2+	27.2	P (3*)	0	0	0	1	1	0	1	1	2	4	0	0	0	0	0	0	0	0	10	Pathogenic (1 str criteria + 1 mod + 4 spp)
	K84E (c.250A>G)	2+	22.5	LP (3*)	0	0	0	2	1	0	1	0	2	4	0	0	0	0	0	0	0	0	10	Pathogenic (1 str criteria + 2 mod + 2 spp)
	R100P (c.299G>C)	2+	0	P (3*)	0	0	0	2	1	0	0	0	2	4	0	0	0	0	0	0	0	0	9	Likely Pathogenic (1 str criteria + 2 mod+ 1 spp)
	**T117M (c.350C>T)**	**0+**	**34.8**	P (3*)	0	0	0	2	1	0	1	4	2	4	0	0	0	0	0	0	0	0	14	Pathogenic (2 str criteria + 2mod + 1spp)
CTD (216-335)	R217C (c.649C>T)	2+	64.8	Conflicting/LB(1*	0	0	0	0	0	0	0	0	0	0	0	-4	0	-2	0	0	0	0	-6	Benign (BS1 and BS2)
	**I219V (c.655A>G)**	**3+**	**60.7**	B (3*)	0	0	0	0	0	0	0	0	0	0	-8	0	-4	-4	0	-1	0	0	-17	Benign (BA1, BS2, BS3 and BP4)
	R226L (c.677G>T)	1+	39.2	LP (3*)	8	4	0	1	1	0	0	1	2	4	0	0	0	0	0	0	0	0	21	Pathogenic (1 vstr + 2 str + 1mod + 3 spp)
	R265C (c.793C>T)	2+	55	P (3*)	8	4	0	2	1	0	0	4	2	4	0	0	0	0	0	0	0	0	25	Pathogenic (1 vstr +v3 str + 2mod + 1 spp)
	E268G (c.803A>G)	2+	78.9	B (3*)	0	0	0	0	0	0	0	0	1	0	0	0	-1	0	0	0	-1	0	-1	VUS (cold. 1 mod + 2 spp) (required for LB: 1 str + 1 spp)
	K286Q (c.856A>C)	2+	78.6	VUS (2*)	0	0	0	0	1	0	0	0	1	0	0	0	0	-1	0	0	0	0	1	VUS (cold) (3 spp, 2 p Pat and 1p B)
	S295G (c.883A>G)	2+	75.5	P (3*)	8	4	0	1	1	0	0	1	1	4	0	0	0	-1	0	0	0	0	19	Pathogenic (1 vstr criterium + 2 strg + 4 spp)
	D304V (c.911A>T)	1+	0	LP (3*)	0	0	0	2	1	0	1	1	2	1	0	0	0	0	0	0	0	0	8	Likely Pathogenic (2 mod criteria + 4 spp criteria)
	H329P (c.986A>C)	1+	25.7	P (3*)	8	0	0	0	1	0	0	0	0	4	0	0	0	0	0	0	0	0	13	Pathogenic (1 vstr + 1 str + 1 spp)
-	A492T (c.1474G>A)	2+	65.3	VUS (1*)	0	0	0	0	0	0	0	0	0	0	0	0	0	0	0	-1	0	0	-1	VUS (cold) only 1 criterium applies
MMR MLH1-CTD (502-756)	V506A (c.1517T>C)	2+	67.6	LP(1*)	0	0	0	0	1	0	0	1	0	1	0	0	0	-1	0	0	0	0	2	VUS (cold) (2 spp P and 1 spp B)
	N551T (c.1652A>C)	0+	78.9	VUS (1*)	0	0	0	0	1	0	0	1	0	4	0	0	0	-2	0	-1	0	0	3	VUS (contradictory criteria)
	E578G (c.1733A>G)	2+	51.2	B (3*)	0	0	0	0	0	0	0	0	0	0	0	0	0	0	0	-1	0	0	-1	Likely Benign
	L588P (c.1763T>C)	1+	68.3	VUS (1*)	0	0	0	0	1	0	0	0	2	0	0	0	0	0	0	0	0	0	3	VUS (1 mod + 1 spp)
	L622H (c.1865T>A)	0+	69.2	P (3*)	0	0	0	2	1	0	2	1	2	4	0	0	0	0	0	0	0	0	12	Pathogenic (1 str + 3 mod + 2 spp)
	R659Q (c.1976G>A)	2+	79.7	VUS (1*)	0	0	0	0	0	0	2	0	1	4	0	0	0	-1	0	0	0	0	6	Likely Pathogenic (1 str criteria + 1 mod + 1 spp_P. 1 spp B)
	T662P (c.1984A>C)	0+	64	LP (3*)	0	0	0	0	1	0	1	0	2	4	0	0	0	-1	0	0	0	0	7	Likely Pathogenic (1 str, 1 mod, 2 spp P, 1spp B)
	E663D (c.1989G>T)	2+	68.5	P (3*)	8	4	0	0	1	0	0	0	0	4	0	0	0	-1	0	0	0	0	16	Pathogenic (1vstr + 2str + 1spp P, 1spp B)
	A681T (c.2041G>A)	0+	69.8	P (3*)	0	0	0	0	1	0	0	4	0	4	0	0	0	-1	0	-1	0	0	7	Pathogenic (2 strong criteria-PP4_str and PP1_str)
	V716M (c.2146G>A)	1+	75.1	B (3*)	0	0	0	0	0	0	0	0	0	0	-8	0	-4	-2	0	0	0	0	-14	Benign (1 vstr BA1. etc...)
	H718Y (c.2152C>T)	2+	84.5	B (3*)	0	0	0	0	0	0	1	0	0	0	-8	0	-4	-2	0	0	0	0	-13	Benign (1 vstr BA1, etc...)

Supplementary Table 2. InSiGHT ClinGen and CanVIG-UK scores attributed to the 31 *MLH1* VUS presented in Table 1. The MLH1 protein domain colors correspond to those in Fig. 2. T117M and I219V (in bold) were used as pathogenic/deficient and benign/proficient MMR activity controls, respectively. "Cold" VUS and "Hot" VUS refer to variants classified with scores between 0 and 2 points, or 4 to 5 points, respectively. Note that two cold VUS were classified either because only one criterium applied, or the combination of applicable criteria did not allow classification as benign. P-Pathogenic, LP- Likely Pathogenic, VUS-Variant of uncertain significance, LB-Likely Benign, B-Benign, mod-moderate, spp-supporting.

Table S3: Comparison of functional assay systems regarding their aim, biological system, read-out, advantages, and limitations. InSiGHT ClinGen and CanVIG-UK classification guidelines attribute high scores to results obtained in cell-free in vitro mismatch repair activity and human cell-based assays

Assay Type or Name	Target molecule	Assay aim	Biological Model	Specific Features	Readout	Advantages	Limitations
Minigene Assay	RNA	To assess the impact of genetic variants on pre-mRNA splicing events	Mammalian cell lines transfected with engineered minigene constructs	Uses cloned genomic fragments containing exons and intronic regions of MMR genes; allows evaluation of the effect of splice-site, exonic or intronic variants	Detection of exon inclusion or skipping by PCR, gel electrophoresis, and sequencing	Direct molecular assessment of splicing defects; independent of patient-derived cells	Engineered reporter system that may not fully reproduce the endogenous gene context or tissue-specific splicing regulation
RT-PCR Analysis	RNA	To evaluate aberrant endogenous splicing events (exon skipping, intron retention), or transcript instability in patient-derived cells	RNA extracted from patient-derived biological material (peripheral blood lymphocytes, hair roots)	Detects naturally occurring transcripts from the endogenous gene; often combined with cDNA sequencing	PCR product size analysis, sequencing, transcript quantification	Technically simple to execute; reflects physiological transcript processing; clinically relevant	Requires fresh patient sample or stored high-quality RNA; low-abundance transcripts may be missed; nonsense-mediated decay (NMD) can limit detection of aberrant transcripts
Cell-free *in vitro* Mismatch Repair Activity (CIMRA) Assay	Protein	To determine functional MMR activity of variant proteins	Cell-free extracts or recombinant proteins	Measures DNA mismatch repair efficiency *in vitro* by joining MMR proteins produced by *in vitro* transcription and translation with engineered mismatch containing plasmids to measure MMR activity.	Quantification of DNA repair activity relative to wild-type controls	Controlled and reproducible experimental conditions to directly measure and quantify MMR repair function	Technically demanding; may not represent the cellular context (e.g. tissue-specific regulatory events, chromatin context)
Yeast Dominant Mutator Effect Assay	Protein	To assess whether MMR variants exert a dominant-negative mutator phenotype *in vivo*	*Saccharomyces cerevisiae* expressing human or corresponding homologous yeast MMR gene variants	Evaluates increased mutation rate caused by defective MMR repair *in vivo*	Growth rates determined by mutation frequency through HOM3 gene reversion or resistance to canvanine	Sensitive functional readout; rapid cell growth kinetics; suitable for studying dominant effects	Yeast biology may not fully reflect mammalian MMR mechanisms
Yeast Two-Hybrid Assay	Protein	To evaluate protein–protein interactions between MMR proteins *in vivo*	*Saccharomyces cerevisiae* cells expressing engineered fusion proteins	Detects direct interaction between two partner proteins, such as MLH1 and PMS2, or MSH2 and MSH6	Reporter gene activation (growth selection, colorimetric or luminescent signal)	Useful for mapping deficient interaction of missense variants; relatively simple and scalable	Measures only interaction between two target proteins at a time, but not functional repair activity
6-Thioguanine Resistance Assay	Protein	To evaluate cellular MMR proficiency through response to thioguanine-induced DNA damage *in vivo*	Hemizygous mouse embryonic stem cells, or human HAP1 or HEK293 cells expressing variant MMR genes	MMR-proficient cells are sensitive to cell-permeable 6-TG, whereas deficient cells survive	Cell viability (replication arrest with subsequent induction of apoptosis)	Functional cellular assay to monitor MMR pathway activity	Requires technically demanding cell line engineering and selection; results may differ with the employed cell lines
Methylation Tolerance-Based Assay	Protein	To assess MMR-dependent DNA damage signaling *in vivo* in response to methylating mutagenic agent	Expression of variant proteins in human MLH1-deficient HCT116, or MSH2-deficient LoVo cells	Uses exposure to methylating agents MNNG to generate DNA damage and test MMR-mediated cytotoxic response	Cell viability (replication arrest with subsequent induction of apoptosis)	Reflects biologically relevant DNA damage response pathways	Technically demanding cell line engineering; cellular response can be influenced by additional DNA repair pathways; interpretation may be complex
